# Hope and hopelessness in the face of ovarian cancer: perspectives from gynecologists

**DOI:** 10.3389/fonc.2026.1931795

**Published:** 2026-08-12

**Authors:** Karen Rosnes Gissum, Caroline Engen, Sigrun Drageset, Ane Gerda Zahl Eriksson, Ingvild Vistad, Roger Strand, Line Bjørge

**Affiliations:** 1 Cancer Clinic, Haukeland University Hospital, Bergen, Norway; 2 Centre for Cancer Biomarkers CCBIO, Department of Clinical Science, Universitetet i Bergen, Bergen, Norway; 3 Neuro-SysMed, Centre for the Study of the Sciences and the Humanities, Universitetet i Bergen, Bergen, Norway; 4 Division of Psychiatry, Haukeland Universitetssjukehus, Bergen, Norway; 5 Department of Health and Caring sciences, Hogskulen pa Vestlandet, Bergen, Norway; 6 Institute of Clinical Medicine, Faculty of Medicine, Universitetet i Oslo, Oslo, Norway; 7 Department of Gynaecological Oncology, Oslo universitetssykehus, Oslo, Norway; 8 Department of Gynaecology and Obstetrics, Sorlandet sykehus HF, Kristiansand, Norway; 9 Institute of Clinical Medicine, Universitetet i Oslo, Oslo, Norway; 10 Centre for Cancer Biomarkers CCBIO, Centre for the Study of the Sciences and the Humanities, Universitetet i Bergen, Bergen, Norway; 11 Department of Obstetrics and Gynecology, Haukeland Universitetssjukehus, Bergen, Norway

**Keywords:** communication, comprehension, disease, gynecologists, illness, ovarian neoplasm

## Abstract

**Background:**

Effective communication plays a key role in fostering strong relationships between patients and healthcare providers during cancer treatment and care. Women with ovarian cancer live with the illness and disease aspects of the condition, while biomedical knowledge tends to focus on the disease. We aimed to gain an understanding of the patient-provider relationship in ovarian cancer from a gynecologists’ perspective.

**Materials and methods:**

We collected data using semi-structured interviews and applied a phenomenological approach to data analysis.

**Results:**

We conducted individual interviews with nine gynecologists between October 2022 and February 2023. Three distinctive features defined the gynecologists’ understanding (1). When interacting with patients, they focused on diagnostic markers and treatment choices, paying more attention to the disease than to holistic well-being (2). The gynecologists’ understanding of ovarian cancer was mainly based on medical knowledge rather than on patients’ experiences (3). The tragic nature of ovarian cancer led the gynecologists to create emotional distance as a means of self-preservation, while harboring a hope that medical advancements would lead to a cure in the future but acknowledging their feelings of helplessness in treating these patients.

**Conclusion:**

Gynecologists understand ovarian cancer from a disease perspective of health. Their role is to maintain the biomedical dimension of condition, upholding patients’ hope as well as their own for medical advancement throughout the ovarian cancer trajectory. Furthermore, gynecologists understand their role as constrained by a rigid system that limits their ability to best provide holistic care to patients.

## 1 Introduction

Cancer incidences are increasing worldwide (1), creating a significant challenge for healthcare systems and providers. This has created a demand for more personalized and precise diagnostics, prognostics, and treatments (2), reflected in rapid adaptations in organizations and practices (3, 4). There has been increasing interest in understanding the experiential aspects of cancer treatment and living with the disease, and qualitative research is seen as valuable for enhancing healthcare delivery systems and services (5). Cancer is increasingly understood as both a disease (an organic disorder) and an illness (a subjective state of psychological awareness of dysfunction) (6). Researchers have highlighted the importance of investigating how novel medical technologies and advancing medical practices affect the experiences of individuals living with malignancies (7). In addition, experiencing symptoms and side effects, receiving a diagnosis and prognosis, adapting to the role of patient, managing information and expectations, and navigating emotions like hope and despair are all integral parts of the illness journey (8).

Qualitative research highlights illness as relational, shaped by experiences of recognition, understanding, invisibility, and neglect. It underscores how others’ reception—knowledge perspectives and actions—shapes the illness experience (9, 10). Healthcare providers’ perspectives and attitudes significantly impact the cancer experience (11). However, there remains a limited understanding of how these perspectives evolve in a rapidly changing medical landscape. While there is a considerable body of research addressing the lived experience of cancer patients (12–15), there are, to the best of our knowledge, few if any studies exploring how the lived experience of cancer is understood and integrated by specialist physicians.

Ovarian cancer is one of the most deadly gynecological cancers in the Western world (1). Despite significant advances in cancer treatment, the prognosis for ovarian cancer remains poor, and less than half of those diagnosed are cured. Recent qualitative studies on ovarian cancer have underscored the profound emotional and psychological challenges accompanying the physical symptoms of the disease, particularly in response to significant bodily changes following intensive treatments such as chemotherapy and surgery (16, 17). Living with ovarian cancer is often likened to a battle, prompting shifts in lifestyle and outlook while navigating feelings of regret and despair about potential outcomes (17). The impact of ovarian cancer on daily life can be so profound that achieving a sense of normalcy becomes complex, leading affected individuals to navigate life moment by moment (18). Our recent study revealed that women with ovarian cancer feel that their challenges and concerns are inadequately addressed or understood by healthcare providers (19). To gain an understanding of the patient-provider relationship in ovarian cancer from a gynecologists’ perspective.

## 2 Materials and methods

### 2.1 Study design

We conducted a qualitative descriptive study with semi-structured individual interviews with open-ended questions.

### 2.2 Participants

The participants were recruited from four different hospitals in Norway. The inclusion criteria were as follows: The participant should be a gynecologist or gynecologist in training and be involved in the diagnosis and treatment of gynecological cancer patients in a hospital setting. In Norway, gynecologic oncology is not a separate specialty, but is practiced by gynecologists with subspecialized expertise in the diagnosis and treatment of gynecological cancers at specialized university hospital cancer centers, where ovarian cancer care is centralized. Information about the study and contact information were sent to gynecologic oncology treatment units at local and university hospitals in Norway. Interested individuals were encouraged to contact the first author by mail or telephone.

### 2.3 Data collection

Interviews were conducted between October 2022 and February 2023. The first author (KRG), an oncology nurse with extensive clinical experience in gynecological cancer care and many years of experience as a patient pathway coordinator for women with gynecological cancers, conducted all interviews, conducted all interviews. One interview was conducted face-to-face, while the others were conducted using a synchronously computer-mediated communication platform with audio and video. The interviews lasted 40–65 minutes and were conducted in Norwegian.

After the ninth interview, we observed that very few new issues were emerging in the dataset. Accordingly, it was concluded that theoretical saturation had been reached. Guided by the principle of data saturation, we continued interviewing until new information became scarce. After the ninth interview, we observed only limited emergence of new issues and therefore considered theoretical saturation to have been achieved (20). The interviews were audiotaped, downloaded, securely stored, and transcribed verbatim.

### 2.4 Data analysis

The data analysis was inspired by Giorgi’s descriptive phenomenological method (21). The authors (KRG, CBNE, SD, and RS) all read each transcript several times to capture the entire content. Then, with an open mind and phenomenological attitude, separately and then together, the authors identified meaning units in the transcripts. A stage of careful consideration began to transform the meaningful units into secondary-order descriptions. The discussions on what constituted meaningful units and the relationships between them went back and forth and were tested among the authors. Giorgi describes this segment as the very core of the analysis, and it was during this step that the participants’ descriptions of their understanding of living with ovarian cancer became visible. In the end, we strived to find structures of the whole material before achieving a final confirmation of the results.

The quotes were translated into English by the first author and verified by the co-authors and a professional translator. Finally, external validation was conducted by three experienced authors (AGZE, IV, and LB), all gynecologists working in oncological settings, thereby ensuring the robustness and credibility of the results. Neither was involved in the data collection or the analysis process.

### 2.5 Rigor

We sought to ensure methodological rigor through reflexive and transparent research practices throughout the study. The first author, who conducted the interviews, had extensive clinical experience working with women diagnosed with ovarian cancer. To minimize the influence of pre-understandings, we adopted a reflexive and phenomenological approach, balancing professional expertise with a stance of qualified naïveté. The interview guide was developed and discussed within the multidisciplinary author group that included gynecologists (LB, AGZE, IV) other health care professionals (CE, SD) and also a philosopher of science (RS) and was closely linked to the study aim. This process helped balance pre-understandings, while the semi-structured format allowed for follow-up questions and exploration of participants’ experiences in greater depth. Throughout the analytical process, interpretations and analytical decisions were continuously discussed among the authors to promote transparency and reflexive interpretation of the data.

### 2.6 Ethics

Study approval was granted by the Norwegian Centre for Research Data on June 17, 2022 (ID no: 887555) prior to the onset of the project. The participants were informed of their right and ability to withdraw from the study at any time without consequences. All participants signed a study-specific informed consent form before their interviews began. An Ethics Submission Assessment submitted to the Regional Committee for Medical and Health Research Ethics Western Norway (ID no: 727498) determined that the absence of health data collection obviated the necessity for an application as per their evaluation.

## 3 Results

### 3.1 Sample characteristics

Nine female gynecologists were interviewed. Both female and male gynecologists working in gynecological units were invited to participate. However, only female gynecologists responded positively to the invitation and were included in the study. Their mean age was 41 years (age range: 30–56 years). Most participants were specialists working at university hospitals. The participants had an average of 12 years (range: 4–22 years) of clinical experience in gynecology.

### 3.2 Constituents emerging from qualitative analysis

#### 3.2.1 Gynecologists’ interactions with their patients: a defined role and a clinical focus

The participants described their interactions with patients as consisting of routine consultations loaded with tasks related to clinical examinations, evaluations of performance status, clinical evaluations of the disease, and decisions regarding further treatment.

It’s often quite short follow-up appointments that are scheduled. We might need to review CT scans, blood tests, side effects, and come up with a further plan. We need to conduct an examination, a physical check, and an ultrasound, all in 30 minutes, so I don’t always believe that discussing what’s important for the patients is given enough focus.

The communicative content of the interaction consisted of conveying factual information, such as objective findings, diagnostic outcomes, prognosis, treatment decisions, and clinical guidelines, with little else discussed.

You’re supposed to provide information about surgery and what it entails, and then you’re supposed to provide information about chemotherapy, and then you’re supposed to provide information about the disease itself.

The purpose of providing information was to fulfill the patients’ rights and the physicians’ corresponding duties concerning patient autonomy and shared treatment decisions, in line with existing clinical guidelines and based on objective clinical information.

I believe the healthcare system is structured (…) so that we should discuss treatment, that’s our concern. There is limited choice in terms of what we should do, and what we can do that aligns with the best medical practice and adheres to the clinical guidelines.

Given the pre-determined organization of the clinical work and the legal and regulatory demands for evidence-based medicine, they usually did not find time to talk with their patients about the experiential or existential dimension of their condition. Specifically, some participants reported that oncological nurses were the ones who should and would take care of the patients in that regard, and not the gynecologists themselves.

You have to maintain a certain professional distance, and in a way, pull yourself together, because you must be the one who remains objective and provides medically reasoned advice. You’re not the psychologist, but you’re in an acute situation as a doctor, and I have looked them in the eyes while delivering the news.

When the gynecologists encouraged patients to share their experiences with ovarian cancer, such as by inquiring about their coping mechanisms, it often led to a flood of words. One participant likened this verbal outpour to “turning on the faucet,” suggesting a continuous stream that would be difficult to stop. However, due to time constraints during clinical appointments, the conversation had to be redirected to ensure that patients understood and consented to proposed treatment decisions and the accompanying biomedical information. Participants perceived their role as primarily managing the biomedical aspects of the disease and ensuring the quality of treatment decisions based on biomedical evidence.

Because we present it [the treatment] as a package, that’s what’s recommended to be done: the combination of both surgery [and] chemotherapy.

#### 3.2.2 Gynecologists’ understanding of ovarian cancer: a tragic reality

Some participants portrayed ovarian cancer by providing an overview of the common adverse effects of ovarian cancer treatment, particularly the physical adverse effects. Others shared thoughts and reflections on how a life with ovarian cancer is likely to be. Several participants described what they considered to be the tragic nature of the condition: the progressive degeneration of the body, the lack of a cure, and the fatal outcome.

They [some patients] have suffered greatly over a long period, and we think that it’s actually the best thing [to end the treatment], because we see that they have endured an uncomfortable, painful, and exhausting treatment where they haven’t had any quality of life, couldn’t enjoy their family and grandchildren, and all the things they haven’t been able to do, what they really desire.

This is not to imply that the participants lack empathy for the severity of the condition or the suffering of the patients.

We know that they won’t get any better from it; at best, it’s life-prolonging treatment. None of the treatments we provide after they’ve had a relapse are curative. It’s just, essentially just extending life, and the life-prolonging treatment comes at a price.

This seemed to provoke a profound sense of hopelessness and helplessness in all participants. Rather than the immediate experience of illness, the primary source of suffering was implicitly attributed to the incurable nature of the disease. The narratives, however, seemed to derive more from the interpreters’ own medical observations than from direct communication with patients regarding their experiences. In fact, the participants described the need or even duty to maintain and communicate hope to the patients in the presence of a poor prognosis and the prospect of a fatal outcome.

We shouldn’t crush the patients by being too brutally honest in our messages, either. It’s important that they receive information they need about their disease, but it’s also important that they retain their hope to be able to endure cancer treatment and to understand that maybe hoping to fully recover entirely is unrealistic, but that they can live for a long time with this as a chronic disease, that’s a possibility.

In fact, the participants identified their professional roles as centered on the crucial responsibility to foster and sustain hope in their patients, refraining from any actions that might diminish hope.

It’s much easier to provide treatment than to stop. It’s always because in doing something, you feel like you’re accomplishing something as a doctor, you want to do something, you want to fix something, you can’t bear to be powerless.

Some participants reported a personal need to maintain a belief in the potential of a future cure for their own work to hold significance.

The emotional impact it has on us—such desperation of everyone, for us and, of course, for the patients, for the entire family—we just hope that we can achieve something.

Some participants also framed hope within the medical context as technological optimism, embodying positive expectations linked to rapid advancements in medical technologies and anticipated breakthroughs in future therapeutic interventions.

We must hold on to hope, completely irrespective of the treatment options available, no matter how badly things are going.

#### 3.2.3 Gynecologists’ self-understanding: the ambiguous role of hope

Several participants articulated the necessity to create some emotional distance and protective boundaries from the intensity of their patients’ situations, aiming to protect their own emotional well-being.

To survive in this industry, you need to have the ability to not get too pulled down by it, and to not take other’s sorrow home with you.

This sentiment was accompanied by a recognized necessity to cultivate a degree of detachment from the emotional experiences of patients. The participants found that the structure of their work facilitated this detachment, as the existential and experiential dimensions were primarily perceived as the responsibilities of nurses, while treatment decisions were predominantly based on biomedical and other objective information that did not directly address the nuances of pain or other forms of experienced suffering.

Several of the participants nevertheless mourned a lack of time and opportunity to adequately address patient concerns and extend empathy, pointing to the constraints of their tightly organized work schedule. Some participants elaborated on the profound impact of navigating these situations in their profession, acknowledging the potential for distress and a sense of helplessness.

It’s not like we can manage [delivering bad news]; we are not machines. We talk to the patients far too little about what you [the interviewer] and I have discussed now.

In the interviews, amid the ambiguity of detachment and engagement, hope and realism, emerged reflexivity. The participants voiced reflections with regard to their professional identities and personal realities. Some expressed existential gratitude for their life and health.

Working with women [with ovarian cancer] has affected me as a person, and perhaps that’s the best part of this job: that you value life and don’t take it for granted, that you are alive and that you have birthdays and that you have healthy children. So, I’m not upset when I have a birthday; it’s enjoyable.

Several participants described experiences of pain, pointing to their own embeddedness and productive role in a healthcare system in which they had lost faith.

I become a representative of a system that doesn’t work. In a way, I become the ambassador who has to be accountable, and I find that to be one of the worst things.

## 4 Discussion

Research on the experiences of ovarian cancer has highlighted the intricate realities that women face, including existential uncertainties, identity disruption, and challenges in communication with healthcare providers, indicating unmet care expectations (19, 22, 23). This study addresses this gap by examining the perspectives of gynecologists in oncological settings. It reveals three interconnected themes: 1) Gynecologists primarily focus on disease assessment, treatment decisions, and information dissemination within their defined role. 2) They perceive ovarian cancer as a tragic disease, and experience feelings of helplessness and hopelessness due to limited technoscientific tools and institutional constraints. 3) Amid technological limitations, hope serves as a tool for meaningful engagement, prompting reflection on professional identity and constraints.

In this material, the informants provided limited descriptions of and engagement with ovarian cancer as a lived experience. This echoes the gap experienced by women with ovarian cancer between expectations and needs of care and current clinical practices (19). Women diagnosed with ovarian cancer expect physicians to actively contribute to their overall well-being and to assist in navigating their illness experience (24). However, this material suggests that gynecologists in oncological settings perceive their roles differently. The participants’ descriptions of their roles and the patient–gynecologist relationship highlight the defined and delimited tasks and responsibilities of gynecologists in this context. Embedded within a larger healthcare system, and operating in collaboration with other healthcare providers, gynecologists are experts of the (bio)medical aspects of the disease, the related medical technologies, and surgical and medical interventions. As such, the patient–gynecologist relationship, as experienced and narrated by the gynecologists, reflects developments in the organization of healthcare delivery systems at large: specialization and differentiation of labor at the level of the system, and shifts in knowledge, skills, tasks, and responsibilities at the level of the various human actors working within these systems. For gynecologists and other specialists, this involves a gradual transition from direct involvement in patient treatment to the increasing delegation of tasks to others while offering supervision and science-based quality assurance.

Task shifting, in the sense of rearranging who does what, is a strategy of rationalization and effectivization of healthcare delivery (25). This raises the question of whether the gap in needs and expectations experienced by women with ovarian cancer should be regarded as a problem. This could be reduced to the question of adjusting and reorienting expectations. Holistic care may still be possible by placing the responsibility elsewhere (e.g., general practitioner, coordinating oncological nurse, or nurse practitioner). However, this material brings attention to fundamental inquiries about the nature of good medical practice and its requirements. Though beyond the scope of this study, participants acknowledged their role as decision-makers in diagnostic, prognostic, and therapeutic matters, encompassing sequencing, intensity, timing, and intervention discontinuation. They recognized the ability to make such decisions without exhaustive knowledge of the illness or the individual.

This is evident in the sparse representation of certain themes in the material. Clinical presentations and symptoms, as well as personal illness narratives, are notably lacking. There are few descriptions of the physical realities of bodies altered by disease, treatments, and surgeries, as well as limited accounts of individuals’ personal stories, complaints, concerns, preferences, priorities, and dreams. Eyal et al. (26) suggests that as a consequence of current developments in healthcare organization, physicians’ roles might be reduced to just one of many partners alongside patients (27). Scholars have argued that this development has already led to a change in physicians’ work modes. A once-focused engagement of clinical signs and symptoms is replaced by an emphasis on measures and metrics and evidence-based practice (28). Despite ever more data and scientific knowledge, however, cancer trajectories involve uncertainty related to treatment choices and outcomes (29). The relationship between medical examination, radiological images, biomarkers of disease, and the treatment and care needs of illness are complex. An overreliance on measures and metrics can result in poor care and poor outcomes (30). This can also contribute to overlooking the patient’s active role by assuming they are merely “passive recipients of treatment, although cooperation with treatment is expected” (31, pp. 1398). Shared decision making and good clinical decision making require the leveraging of experiences and values. To do so, physicians would have to collect clinical information beyond the disease-focused data to obtain a broader understanding of the patient’s overall illness experience (32), preferences, and priorities. The types of knowledge and relationships required for shared decision-making involve the expansion and externalization of disease-related knowledge, as well as the division of narrative and experiential knowledge.

The experiences of hopelessness and helplessness, along with gynecologists’ perception of ovarian cancer as a tragic disease, imply that changes in practices and responsibilities may affect gynecologists themselves. The limitations of accessible knowledge for clinical decisions not only diminish gynecologists’ understanding but also hamper their connection to patients. This narrowing is closely linked to the recurring theme of hope that emerged in the interviews. Hope was grounding, serving both as a necessary illusion and as a potential driver of future medical breakthroughs. Hope provided connection, community, and communion, anchoring the gynecologist in a relational space with their patients where they had little else to offer, as well as connecting the gynecologist with their own disciplinary practice, which had little else to offer them. Hope emerged as sense-making and meaning-making in a kind of practice that had been hollowed out. Hope, however, appeared both fragile and ambivalent, encompassing elements of false hope in relation to patients and technological advancements. This ambivalence emerged as a productive aspect in the interviews, fostering reflexivity regarding disciplinary practice and its interaction with broader structures. Gynecologists, in grappling with technoscientific constraints and institutional pressures, invoke a fragile hope as both a remedy for patients and an emotional defense for themselves. While holistic care is a recognized concept in healthcare, different professions exhibit varied approaches to its implementation (33).

It should be noted that all participants in the present study were female gynecologists. Gender may influence professional perspectives and approaches to patient care. Consequently, the findings should be interpreted as an in-depth exploration of female gynecologists’ perspectives rather than being assumed to represent gynecologists more broadly.

The types of knowledge and relationships required for shared decision-making involve the expansion and externalization of disease-related knowledge, as well as the division of narrative and experiential knowledge. This study demonstrates how specialized and differentiated healthcare delivery systems and the human actors who inhabit them struggle with holism and the fragmentation of care relationships. The gap could be bridged by allowing physicians to function as “doctors-as-persons” with emotions and affect (34, 35), with time, and with a mandate to listen to and act upon patients’ experiences of illness (36). This could enhance treatment decisions, improve care quality, and alleviate emotional distress for patients with challenging conditions like ovarian cancer. Additionally, it may help alleviate moral stress and emotional burdens for physicians.

### 4.1 Study limitations

This study has certain limitations, particularly in terms of participant selection. The majority of the participants were affiliated with Norwegian university hospitals, and all were female. It is unclear whether our findings can be generalized to other contexts, such as gynecologists practicing in local hospitals. A more diverse sample including both female and male gynecologists might have resulted in different perspectives and experiences being represented. It should be noted, however, that in the Norwegian context, patients with ovarian cancer are highly likely to be met and treated by female gynecologists at major hospitals, also as the percentage of females among Norwegian gynecologists is high and rapidly increasing.

### 4.2 Implications for practice

The current practice in healthcare poses potential risks, such as emotional burnout among physicians and an elevated likelihood of anxiety and depression among cancer patients, as their comprehensive experiences of illness often go unaddressed. However, these consequences are not inevitable and can be mitigated. Gynecologists themselves suggest that fostering continuity in the patient–physician relationship could enhance communication and provide a deeper understanding of patients’ needs, ultimately contributing to the delivery of optimal possible care and treatment for both their illness and disease experiences.

### 4.3 Conclusion

Together, the presented data provides insights into the interplay between the existential and personal dimensions of the participants’ lives and their responsibilities and roles as gynecologists. Our findings indicate that gynecologists understand ovarian cancer as a disease rather than an illness. Their comprehension is shaped by institutional frameworks and protocols that prioritize disease-centric approaches in healthcare systems, constraining their ability to provide comprehensive services tailored to the diverse needs of patients. These constraints impede communication between patients and gynecologists, which could offer valuable insights into illness and wellness and potentially enhance cancer treatment and care. These findings are relevant for clinical practice, as they illuminate how the predominant emphasis on disease and biomedicine influences interactions between physicians and patients.

These findings are relevant for clinical practice, as they illuminate how the predominant emphasis on disease and biomedicine influences physician-patient interactions and may limit opportunities to address patients’ existential and personal concerns. The findings further suggest that these challenges may contribute to the substantial emotional burden experienced by physicians caring for women with advanced ovarian cancer.

## Data Availability

The original contributions presented in the study are included in the article/supplementary material. Further inquiries can be directed to the corresponding author.
